# Constructing Scissor+ risk model to predict prognosis and immunotherapy responses in PAAD by integrating bulk and single-cell RNA sequencing data

**DOI:** 10.3389/fphar.2025.1646840

**Published:** 2025-09-19

**Authors:** Gaofei Zhang, Jiao Yu, Fan Zhang, Fang Wang, Congya Zhou

**Affiliations:** ^1^ Department of Radiation Oncology, Shaanxi Provincial People’s Hospital, Xi’an, Shaanxi, China; ^2^ Department of Oncology, Shaanxi Provincial People’s Hospital, Xi’an, Shaanxi, China

**Keywords:** Single-cell RNA sequencing, PAAD, Scissor, Epithelial cells, Prognosis, Tumor microenvironment

## Abstract

**Background:**

This study focused on epithelial cells to construct a prognostic risk model and provide targeted insights into responses to immunotherapy.

**Methods:**

Single-cell RNA sequencing (scRNA-seq) was clustered using Uniform Manifold Approximation and Projection (UMAP) and a risk model was developed through Least Absolute Shrinkage and Selection Operator (LASSO) regression analysis. Kaplan-Meier analysis was performed to evaluate the prognosis of PAAD. The biological characteristics of LIPH were assessed using CCK-8, colony formation and Transwell assays.

**Results:**

Eight major cell clusters were identified, revealing two developmental trajectories for malignant epithelial cells from primary to metastases. Epithelial cells were categorized into Scissor+ and Scissor- subtypes, with Scissor+ epithelial cells exhibiting more complex cellular communication with TME cells. Furthermore, we successfully developed a risk model for PAAD patients based on the Scissor findings. The prognosis for PAAD patients in the high-risk group was significantly poorer within both the TCGA and ICGC cohorts. Differences were observed in the populations of naïve B cells, CD8 T cells, M0 macrophages, and activated dendritic cells in different groups. Knockdown of LIPH significantly inhibited the growth and invasion of PAAD cells.

**Conclusion:**

These findings underscore the significance of this risk model in predicting prognosis and immunotherapy responses, and enhancing understanding of tumor microenvironment (TME) heterogeneity in PAAD metastases.

## 1 Introduction

Pancreatic adenocarcinoma (PAAD) is among the deadliest human malignancies and is projected to become the third leading cause of cancer-related mortality by 2025 (Siegel et al., 2024; Bray et al., 2024; Stoop et al., 2025). The majority of PAAD patients have been diagnosed with distant metastases, and approximately 80% ultimately succumb to these metastases (Park et al., 2021). Although chemotherapies such as NALIRIFOX, a modified FOLFIRINOX, and gemcitabine combined with nanoparticle albumin-bound paclitaxel have significantly improved overall survival (OS) for metastatic PAAD patients, drug resistance typically develops within 6 months. Furthermore, the median OS for metastatic PAAD patients is less than 12 months (Von Hoff et al., 2013; Hu et al., 2024; Conroy et al., 2023). A critical factor contributing to this situation is the complexity and heterogeneity of cancer cells and the TME. Therefore, there is an urgent need for comprehensive exploration of the heterogeneity of both cancer cells and cancer-related cells within the tumor microenvironment (TME) to inform therapeutic decisions for patients with metastatic PAAD.

The TME of PAAD is a highly heterogeneous system composed of tumor cells, endothelial cells, cancer-associated fibroblasts (CAFs), immune cells, and the extracellular matrix (Han et al., 2021; Grunwald et al., 2021). The TME of PAAD plays a crucial role in tumor initiation, metastasis, metabolic reprogramming, immune evasion, and drug resistance (Raghavan et al., 2021). With advancements in high-throughput sequencing technologies, single-cell RNA sequencing (scRNA-seq) has emerged as a powerful tool for elucidating the heterogeneity of the TME in PAAD, allowing for the exploration of distinct cell subsets and the dissection of developmental trajectories among various subpopulations at the single-cell level (Fan et al., 2022; Moncada et al., 2020; Schalck et al., 2022). Lin et al. utilized scRNA-seq to identify the heterogeneity of cellular compositions in primary and metastatic PAAD tumors (Lin et al., 2020). Additionally, Zhang et al. revealed that subpopulations of CD103+PD-1+CD39^+^ T cells and CD73^+^ macrophages could promote liver metastasis in PAAD (Zhang Z. et al., 2023). PAAD originates from epithelial cells, and Kim et al. identified a novel epithelial cell cluster, demonstrating its prognostic value during cancer progression (Kim et al., 2024). However, the heterogeneity of cancer cell subpopulations, their characteristic gene expression, and their interactions with the TME in PAAD metastases remain poorly understood. Recently, the Scissor algorithm was reported to identify phenotype-associated cell subpopulations from scRNA-seq and bulk RNA-seq data (Sun et al., 2022). This advancement will aid in the comprehensive understanding of metastasis-related subclusters and facilitate the exploration of therapeutic targets for patients with metastatic PAAD.

In this research, we investigated the potential heterogeneity of primary and metastatic PAAD samples at the single-cell level by integrating scRNA-seq and bulk RNA sequencing data. Furthermore, we identified characteristic malignant epithelial cell subsets, referred to as Scissor+ epithelial cell subsets, and constructed a risk model incorporating candidate genes (TATDN1, CAV2, CLDN1, LIPH, MT1E, PSCA, and MMP3). We evaluated the prognostic and immunological implications of this risk model. Notably, we observed that LIPH expression was elevated in tumor tissues of PAAD patients compared to paired adjacent tissues. Additionally, our results indicated a significant reduction in both the proliferation and metastasis of PAAD cells following LIPH knockdown. These findings enhance our understanding of the TME heterogeneity in PAAD metastasis and provide potential prognostic and immunological signatures for PAAD patients.

## 2 Materials and methods

### 2.1 Data acquisition

ScRNA-seq data, comprising 10 primary and six metastatic PAAD samples, were downloaded from the Gene Expression Omnibus (GEO) database, specifically from series number GSE154778 (Lin et al., 2020). Additionally, bulk RNA sequencing gene expression matrices for PAAD, along with corresponding clinical information such as age, gender, and tumor stage, were retrieved from The Cancer Genome Atlas (TCGA) and the International Cancer Genome Consortium (ICGC). The RNA expression data were transformed into transcripts per million (TPM) and subsequently log-normalized. Information regarding single nucleotide mutations, including small insertions and deletions (indels), was acquired via the cBioPortal website (https://www.cbioportal.org/). Furthermore, RNA-seq data for non-small cell lung carcinoma patients treated with anti-PD-1/PD-L1 therapy were obtained from the GEO database under series number GSE135222 (Kim et al., 2020). RNA-seq data for clear cell renal cell carcinoma patients undergoing PD-1 blockade were sourced from a previously published study (Braun et al., 2020).

### 2.2 ScRNA-seq data processing and clustering

The scRNA-seq data were imported into the R package Seurat (Satija et al., 2015). Genes expressed in fewer than 3 cells, cells expressing fewer than 200 genes, and cells containing more than 20,000 or fewer than 1,000 unique molecular identifiers were excluded. Cells with more than 10% mitochondrial gene expression were filtered out. The R package DoubletFinder was utilized to remove potential doublets (McGinnis et al., 2019). We normalized quality-controlled data using the NormalizeData function with default parameters. Based on the natural-log transformed normalized gene expression matrix, 2,000 highly variable genes were identified using the FindVariableFeatures function with the variance stabilizing transformation (VST) method. All genes were scaled using the ScaleData function. Principal Component Analysis (PCA) was performed to reduce dimensionality using the first 2,000 highly variable genes. The first 30 dimensions were employed to cluster cells using the FindNeighbors and FindClusters functions (resolution = 0.8). The Uniform Manifold Approximation and Projection (UMAP) method was applied to further reduce dimensionality based on the top 30 principal components. Cell types were annotated according to their canonical markers.

### 2.3 Identifying malignant epithelial cells

Epithelial cells were extracted both copyKAT (Gao et al., 2021) and inferCNV (Patel et al., 2014) methods were used to identify malignant epithelial cells. Epithelial cell subclusters annotated as diploid by copyKAT and having relative lower copy number variations (CNVs) score calculated by inferCNV were regarded as normal epithelial cells and excluded for subsequent analysis. ScRNA-seq data processing and clustering.

### 2.4 Trajectory inference analysis

To elucidate the trajectory of malignant epithelial cells from primary to metastatic tissues, we first identified differentially expressed genes (DEGs) between the two groups using the FindMarkers function. These DEGs were then provided to the R package Monocle2 for dimensionality reduction via the reduce Dimension function (Qiu et al., 2017). Genes that exhibited changes along the pseudotime were measured and visualized using a heatmap, and these genes were subsequently clustered into subgroups based on their expression patterns.

### 2.5 Scissor analysis

To identify malignant epithelial cells associated with metastasis in scRNA-seq data, we collected clinical information from patients in TCGA-PAAD, categorizing those diagnosed with Stage IV as metastatic. The R package Scissor was employed to reference corresponding bulk assays and estimate cell subpopulations with the highest metastatic potential. Significant DEGs were identified by comparing the populations of Scissor+ and Scissor- cells using the FindMarkers function, applying a screening threshold of p < 0.05 and log2FC >0.25. Additionally, functional enrichment analysis of the DEGs was conducted using Gene Ontology (GO) and Kyoto Encyclopedia of Genes and Genomes (KEGG) pathway annotations through the R package clusterProfiler. ScRNA-seq data processing and clustering.

### 2.6 Cell-cell communication analysis

For comparison of the cell-cell communication between Scissor+, Scissor- malignant cells and other cell types, the R package SingleCellSignalR was used to infer the ligand-receptor (LR) interactions (Cabello-Aguilar et al., 2020). The cut off for confident predicted LR interactions was set as 0.5.

### 2.7 Construction and validation of prognostic Scissor+ risk score model

We first performed univariate Cox regression analysis implemented in R package survival to determine the prognostic value of upregulated DEGs in Scissor+ cells. Unfavorable genes were determined based on the criteria hazard ratio (HR) >1 and p < 0.05. Next, we performed least absolute shrinkage and selection operator (LASSO) penalized cox regression algorithm with ten-fold cross-validation implemented in R package glmnet to fit the scissor + risk score model for predicting OS of patients. We established the risk score model with the following formula: risk score = sum (normalized expression of each significant gene multiplies its coefficient). Subsequently, we used the surv_cutpoint function implement in R package survminer to determine the optimal cut-off and divided patients into High and Low risk groups. For validation, four types of patients’ survival information, namely, OS, disease specific survival (DSS), disease free interval (DFI), and progression free interval (PFI) were used to explore the potential prognostic value of risk score model.

Additionally, we performed the same scoring system to the ICGC dataset and examine the prognostic value of the model. Besides, we used R package timeROC for time-dependent receiver operating characteristic (ROC) analysis of 1 and 3-year survival, and quantifying the area under the curve.

### 2.8 Nomogram construction and evaluation

Univariate and multivariate Cox regression analyses, along with a nomogram incorporating patient variables such as age, gender, tumor stage, and risk score, were employed to comprehensively evaluate the 1-, 3- and 5-year survival outcomes of patients. The accuracy of the nomogram was assessed using a calibration curve based on the Hosmer-Lemeshow test. Additionally, the predictive ability and clinical benefit of the nomogram were determined through decision curve analysis.

### 2.9 Tumor microenvironment estimation

The immune score, stromal score, tumor purity, and ESTIMATE score of individual patients were estimated using R package estimate. Additionally, the proportion of 22 common immune cell types were predicted by conventional marker genes with the help of R package CIBERSORT. TIDE prediction score was obtained from online website (http://tide.dfci.harvard.edu/).

### 2.10 Somatic mutation analysis

R package maftools was utilized to evaluate somatic variant data, including frame shift insertion, frame shift deletion, missense mutation, nonsense mutation, in frame deletion, in frame insertion, splice site, and multi-hit between high and low risk score groups (https://pubmed.ncbi.nlm.nih.gov/30341162/). The tumor mutational burden (TMB) for each patient was calculated as total mutations divided total covered bases*10^6^.

### 2.11 Cell culture and gene knockdown assay

PANC-1, BXPC-3, ASPC-1, CFPAC-1, CAPAN-1, CAPAN-2 and normal human pancreatic duct epithelial (HPDE6-C7) cell lines were acquired form the BeNa Culture Collection (Bejing, China). The HEK293T cells were obtained from the Cell Repository of the Chinese Academy of Sciences (Shanghai, China). All of cells were cultured at 37 °C with 5% CO2 in DMEM, RPMI 1640 or IMDM medium adding with 10% serum, 100 U/mL penicillin and 100 mg/mL streptomycin (purchased from Beyotime, China).

Gene knockdown assay which included vector construction, virus packaging and lentiviral infection was constructed as previously described (Hu et al., 2019). The target sequence for shLIPH#1 was GCC​CAC​ATA​TCT​GGG​TTT​GTT, and the target sequence for shLIPH#2 was GCG​TCC​TAT​GGA​TGT​CAC​ATT.

### 2.12 qRT-PCR assay

Total RNA from different cells was isolated with Cell Total RNA Isolation Kit (FORGENE, Cat# RE-03111) according to the protocol. The extracted RNA was reversed transcription as complementary DNA (cDNA) with PrimeScript™ RT Master Mix Kit (TAKARA, Cat# RR036A). Then the RT-qPCR was performed using Bio-Rad CFX maestro system. The relative mRNA expression was calculated using the 2^−ΔΔCT^ method. The GAPDH gene was a control gene. LIPH forward primer (5′to 3′): CAA​CGG​GAA​ACC​TCA​CCA​AGA​C, Reverse primer (3′to 5′): AGC​CAG​GTT​GAT​CCA​ATC​CTC​C. GAPDH forward primer (5′to 3′): GTC​TCC​TCT​GAC​TTC​AAC​AGC​G, reverse primer (3′to 5′): AC-CAC​CCT​GTT​GCT​GTA​GCC​AA.

### 2.13 CCK-8 and colony formation assay

To assess cell viability following LIPH knockdown, treated cells were seeded into 96-well plates at a density of 2000 cells per well. Subsequently, the CCK-8 reagent was added to each well, and the cells were incubated for 2 h at room temperature. Absorbance at 450 nm was measured using a multifunction microplate reader (BioTeK) at time points of 0, 24, 48, 72, and 96 h. For the colony formation assay, treated cells were plated in 24-well plates at a density of 1,000 cells per well. After a 14-day culture period, the cells were fixed with formaldehyde (Sigma-Aldrich) and subsequently stained with crystal violet (Sigma-Aldrich) for 30 min. The cell colonies were counted after being washed with deionized water.

### 2.14 Transwell assay

Transwell assay was conducted to assess the invasive and migratory effect of the cells. Treated cells with serum-free medium were placed in the upper chamber, while the lower chamber contained 600 μL of medium supplemented with 10% FBS. For invasion assay, Matrigel (BD Biosciences, USA) was added to the upper chambers. After incubated with 48 h, the cells in the upper chamber were removed, and cells mi-grated to the lower chamber were fixed with paraformaldehyde and stained with crystal violet. Subsequently, the cells were photographed for analysis.

### 2.15 Statistical analysis

Statistical analyses were performed using R version 4.0.2 (https://www.r-project.org/). The Wilcoxon rank sum test was employed to assess differences in expression between the two groups. The Log-rank test and Kaplan-Meier curves were utilized to evaluate the survival distributions of PAAD patients. A two-tailed Student’s t-test and one-way ANOVA were conducted to assess differences among the various groups. All data analyses were performed using GraphPad Prism version 6.0, with a significance threshold set at P < 0.05. Results were visualized with R package ggplot2.

## 3 Results

### 3.1 Clustering scRNA-seq data in PAAD patients

**FIGURE 1:**
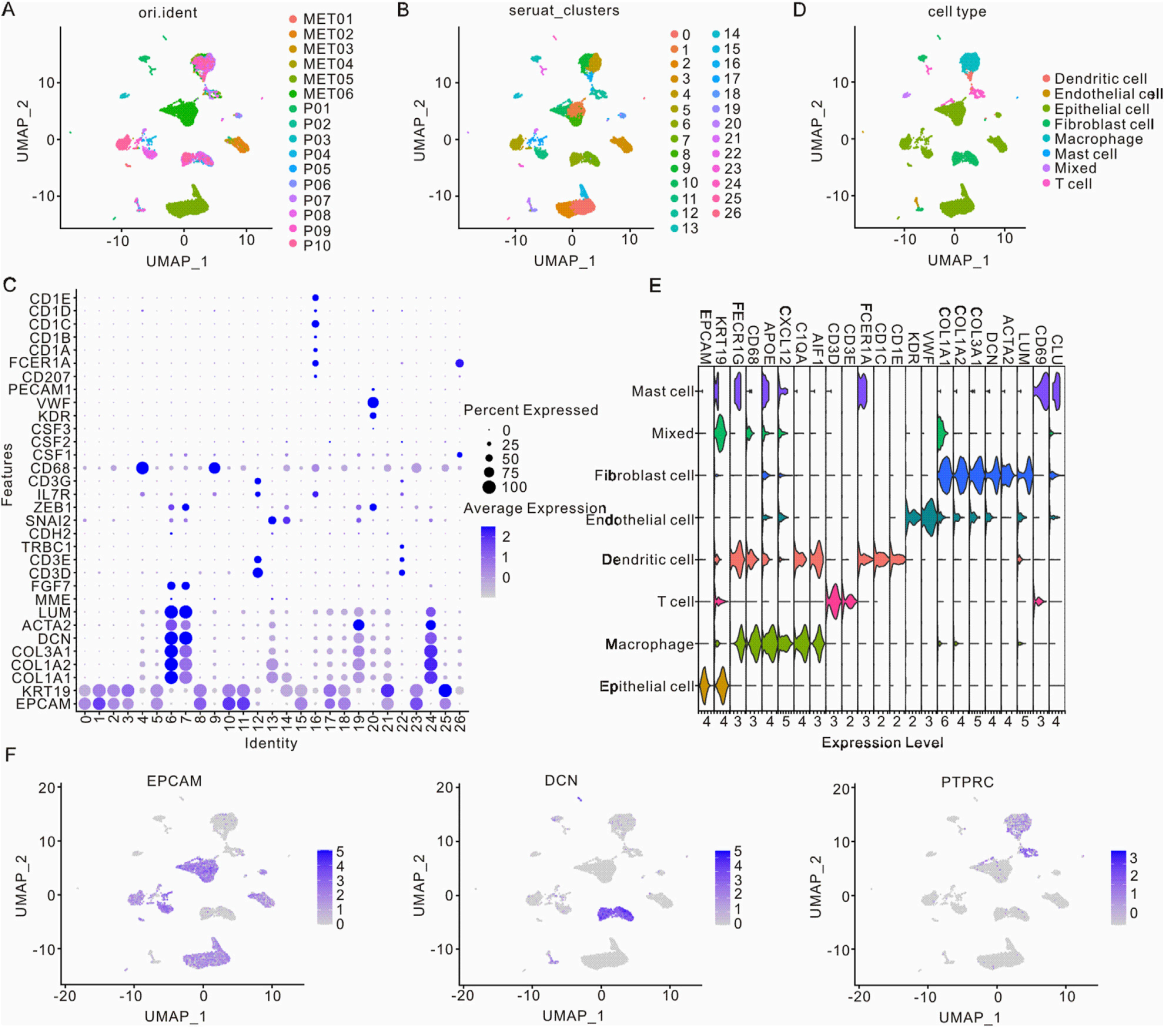
Cell clusters in primary and metastatic samples from PAAD patients. **(A)** UMAP visualization of clusters from various PAAD patients. **(B)** UMAP visualization of PAAD cells differentiated by Seurat cluster coloration. **(C)** Visualization of differentially expressed genes across 27 cell clusters. **(D)** UMAP visualization of eight distinct cell types in PAAD patients. **(E)** Visualization of differentially expressed genes in these eight cell types. **(F)** UMAP visualization of marker genes in epithelial cells, immune cells, and matrix cell types. PAAD, Pancreatic adenocarcinoma; UMAP, Unified Flowform Approximation and Projection.

To comprehensively explore cellular heterogeneity in primary and metastatic samples of PAAD, the scRNA-seq dataset (GSE154778) was downloaded and analyzed. Following normalization and quality control procedures, a total of 11,420 cells and 23,759 genes were obtained from six metastatic samples and 10 primary samples (Figure 1A; Supplementary Figure S1A). Subsequently, twenty-seven clusters were identified through UMAP analysis, with each cluster exhibiting its own uniquely expressed genes (Figures 1B,C). Additionally, t-distributed Stochastic Neighbor Embedding (t-SNE) analysis was also performed to identify cell clusters (Supplementary Figure S1B-E). According to the marker genes, 27 cell clusters were annotated into eight major cell types, including epithelial cells (EPCAM and KRT19), macrophage (AIF1, C1QA, CXCL2, APOE, CD68, and FCER1G), T cells (CD3D and CD3E), dendritic cells (CD1E, CD1C, FCER1A, AIF1, C1QA, APOE, CD68 and FCER1G), endothelial cells (KDR, COL1A1, COL1A2, AOL3A1 and VWF), fibroblast (CLU, LUM, ACTN2, DCN, COL1A1, COL1A2 and AOL3A1), mast cells (CLU, CD69, FCER1A, CXCL2, APOE, FCER1G and KRT19) and mixed cells (Figures 1D,E). The epithelial cells, immune cells and matrix cells were marked by EPCAM, PTPRC and DCN, respectively (Figure 1F; Supplementary Figure S1F).

### 3.2 Identification of epithelial cells in PAAD

**FIGURE 2:**
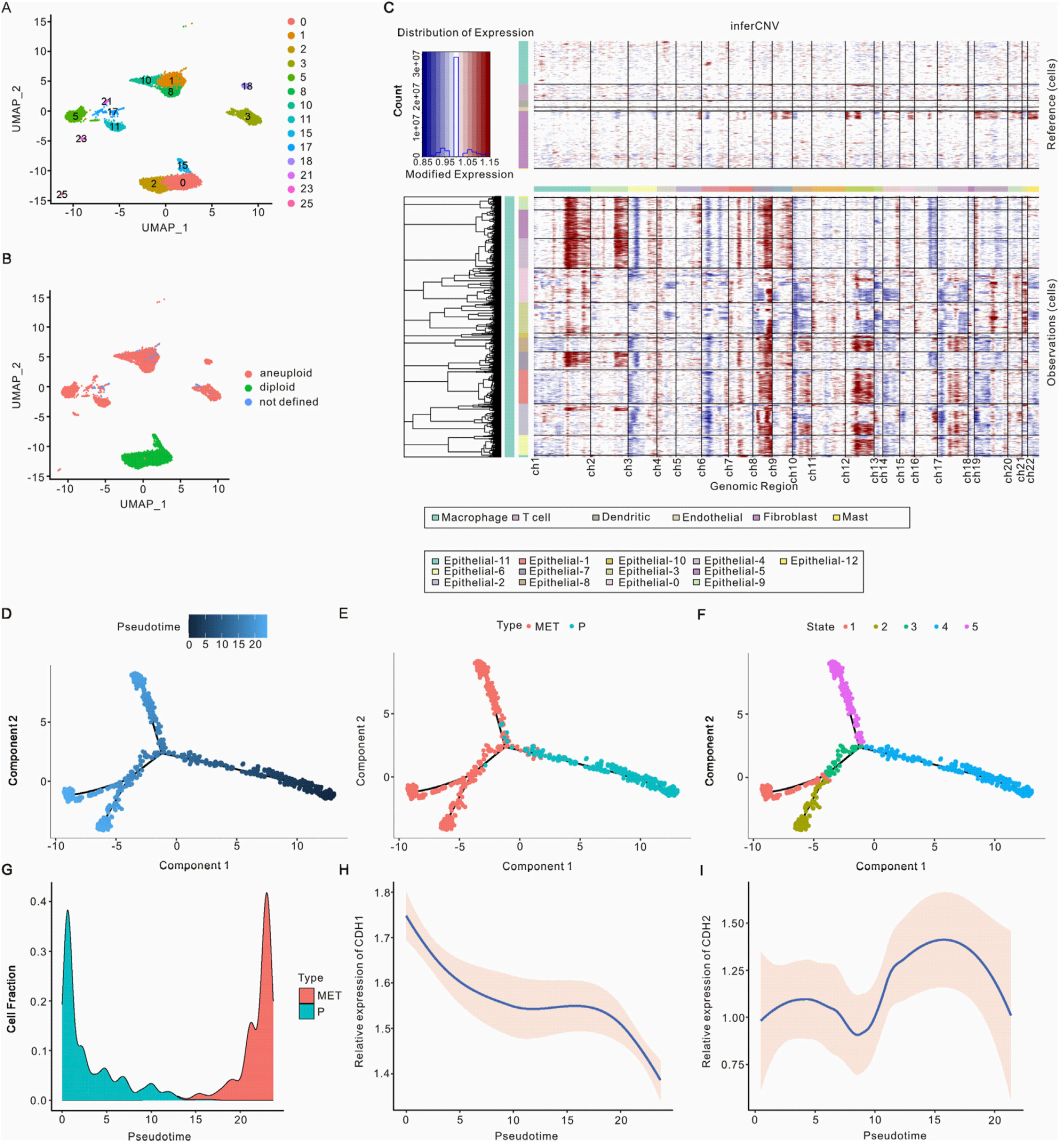
Identification of epithelial cells in PAAD. **(A)** UMAP visualization of epithelial cell clusters in PAAD. **(B)** UMAP visualization of epithelial cell clusters with aneuploid analysis. **(C)** Identification of malignant epithelial cells based on inferred CNVs from various samples, revealing that non-malignant epithelial cells exhibited no significant CNVs. **(D–F)** The differential trajectories of epithelial cells during the progression from primary tumors to metastasis are il-lustrated according to pseudotime, cell type, and cellular state, respectively. **(G)** The proportion of malignant epithelial cells throughout the transition from primary tumors to metastasis. **(H)** CDH1 expression levels in malignant epithelial cells during the progression from primary tumors to metastasis. **(I)** CDH2 expression levels in malignant epithelial cells throughout the evolution from primary tumors to metastasis. PAAD, Pancreatic adenocarcinoma; UMAP, Unified Flowform Approximation and Projection; CNV, Copy number variant.

Since ductal epithelial cells are a population of cells originating from PAAD, we proceeded to further analyze the heterogeneity and potential function of epithelial cells in PAAD. Following UMAP and t-SNE analysis, four major cell types were identified across 14 clusters (Figure 2A; Supplementary Figure S2A). The aneuploidy and diploidy analysis revealed that clusters 0, 2, and 15 were classified as diploid, whereas the remaining clusters were identified as aneuploid (Figure 2B; Supplementary Figure S2B). Furthermore, inferCNV analysis was performed to assess the heterogeneity of epithelial cells. As illustrated in Figure 2C; Supplementary Figure S3A, clusters 17, 23, 11, 3, 21, and 5 exhibited a higher number of CNVs, whereas clusters 0, 2, and 15 showed lower CNVs, corroborating the findings from the aneuploidy analysis. Additionally, we analyzed the developmental trajectories of malignant epithelial cells in both primary and metastatic samples. Pseudtotime analysis, along with cell cluster and state estimation analyses, were employed to investigate the developmental trajectories of these malignant epithelial cells (Figures 2D–F). The results indicated that malignant epithelial cells progress from primary tumors to metastases, suggesting an underlying biological process driving PAAD metastasis.

As the progression from primary tumors to metastasis occurs, the fraction of malignant epithelial cells initially decreases before subsequently increasing. Notably, two distinct developmental trajectories for malignant epithelial cells from primary tumors to metastases have been observed (Figure 2G; Supplementary Figure S3B). We analyzed the expression of the epithelial cell marker gene CDH1 and the mesenchymal cell marker gene CDH2, finding that CDH1 expression decreased while CDH2 expression increased in malignant tumor epithelial cells as the tumor progressed from primary to metastatic stages (Figures 2H,I). These findings are consistent with previous studies and support the reliability of the malignant epithelial cell subsets we identified. Overall, we identified malignant epithelial cells and characterized two developmental trajectories from primary tumors to metastasis. Differential gene analysis revealed that DCBLD2, KLK6, RBP1, LY6D, and RNF43 were expressed at higher levels in metastatic malignant epithelial cells, whereas TFF2, TFF1, CTSE, VSIG2, and CLDN18 were more abundant in primary malignant epithelial cells (Supplementary Figure S3C). Based on the expression of the top 1,000 differentially expressed genes, malignant epithelial cells can be categorized into six distinct clusters (Supplementary Figure S3D). As tumors progress from primary to metastatic stages, clusters 2, 1, and 4 exhibit a decrease, while clusters 5, 3, and 6 show an increase. GO analysis indicated that the top pathways associated with clusters 2, 1, and 4 included oxidoreduction-driven activity, electron transfer activity, molecular function inhibitor activity, viral life cycle, cytoplasmic translation, and regulation of nitric oxide metabolism. Conversely, the top pathways in clusters 5, 3, and 6 encompassed actomyosin, actin filament bundle formation, RNA splicing via transesterification reactions, cytoplasmic translation, cell-substrate junctions, and ribosomal function (Supplementary Figure S3E).

### 3.3 Characteristics and intercellular communication of Scissor+ epithelial cells in metastases

**FIGURE 3:**
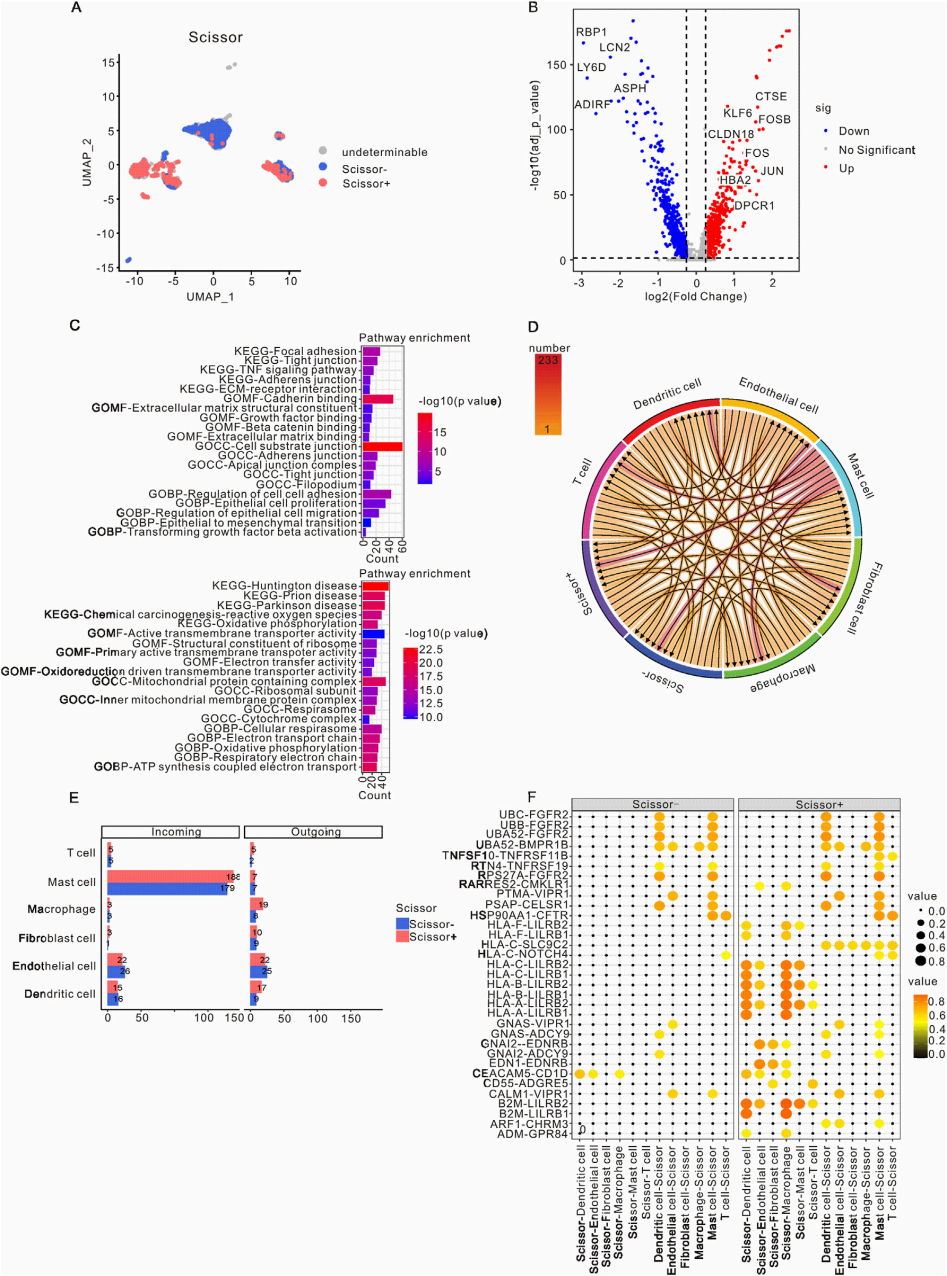
Characteristics and intercellular communication of Scissor+ epithelial cells in PAAD patients. **(A)** A UMAP visualization highlights the epithelial cells in PAAD samples, with Scissor+ epithelial cells indicated in red and Scissor- epithelial cells in blue. **(B)** The volcano plot presents differential gene expression between Scissor+ and Scissor- epithelial cells, where red represents genes that are upregulated in Scissor+ epithelial cells, and blue denotes genes that are downregulated in comparison to Scissor- epithelial cells. **(C)** GO and KEGG analyses were performed on the upregulated and downregulated genes in Scissor+ epithelial cells relative to Scissor- epithelial cells. **(D)** The intercellular communication network within the TME is depicted. **(E)** The incoming and outgoing signaling patterns of the Scissor+ and Scissor- groups are presented. **(F)** CellCall analysis elucidates the distinct communication pathways between Scissor+ and Scissor- epithelial cells. PAAD, Pancreatic adenocarcinoma; TME, Tumor microenvironment; UMAP, Unified Flowform Approximation and Projection; GO, Gene Ontology; KEGG, Kyoto Encyclopedia of Genes and Genomes.

To identify subclusters of malignant epithelial cells associated with metastasis, we employed the Scissor algorithm to integrate scRNA-seq data with bulk RNA-seq data. As illustrated in Figure 3A and Supplementary Figure S2C, the malignant epithelial cells were categorized into Scissor+ epithelial cells, Scissor- epithelial cells, and undetermined epithelial cells. Notably, there was a higher prevalence of Scissor+ epithelial cells in primary samples compared to Scissor-epithelial cells, which were more abundant in metastatic samples (Supplementary Figure S4A). A total of 741 genes were found to be upregulated, while 793 genes were downregulated in Scissor+ epithelial cells when compared to Scissor- epithelial cells. Among the upregulated genes were KLF6, CTSE, FOSB, CLDN18, FOS, JUN, HBA2, and DPOR1, whereas downregulated genes included RBP1, LCN2, LY6D, ASPH, ADIRF, and ASPH (Figure 3B). GO functional enrichment analysis indicated that the upregulated genes in Scissor+ epithelial cells were primarily involved in cadherin binding, cell-substrate junctions, and the regulation of cell-to-cell adhesion. In contrast, the downregulated genes in Scissor+ epithelial cells were associated with structural constituents of ribosomes, ribosomal subunits, and the respiratory electron transport chain (Figure 3C). The KEGG pathway analysis revealed that the upregulated genes in Scissor+ epithelial cells were enriched in pathways related to focal adhesion, tight junctions, TNF signaling, and ECM-receptor interactions, which are characteristic of tumor metastasis (Figure 3C). Conversely, the downregulated genes were predominantly involved in prion disease, Parkinson’s disease, oxidative phosphorylation, Huntington’s disease, and chemical carcinogenesis related to reactive oxygen species (Figure 3C). Additionally, we observed that the overexpressed genes in the Scissor+ group were enriched in epithelial-mesenchymal transition (EMT) and Transforming growth factor β (TGFβ) signaling pathways (Supplementary Figure S4B).

The cell-cell communication network was utilized to assess the communication probability of Scissor+ and Scissor- epithelial cells with other TME cells using CellChat. The analysis revealed that Scissor+ epithelial cells exhibited a higher level of communication with various TME cells, with the most prominent interactions occurring with mast cells, endothelial cells, dendritic cells, and macrophages (Figures 3D,E). The specific pathways through which Scissor+ and Scissor- epithelial cells interacted with other TME cells are detailed in Supplementary Figure S5. Notably, compared to Scissor- epithelial cells, Scissor+ epithelial cells engaged with mast cells, endothelial cells, dendritic cells, and macrophages via the HLA-LILRB and B2M-LILRB pathways (Figure 3F).

### 3.4 Construction of prognostic risk model for PAAD

**FIGURE 4:**
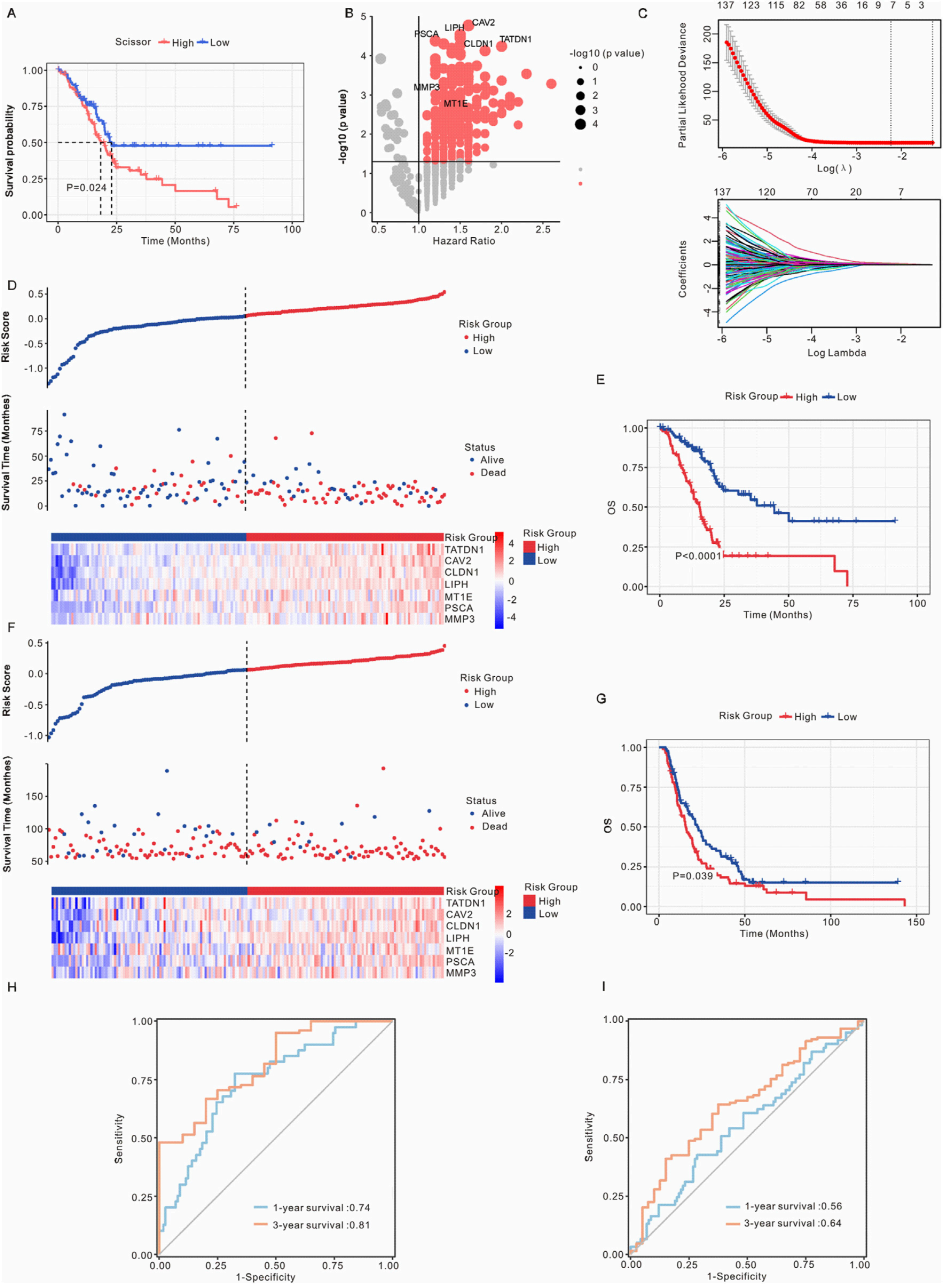
Establishment of the prognostic risk model. **(A)** Kaplan-Meier analysis of survival probabilities for PAAD patients in the Scissor+ and Scissor- epithelial cell groups. **(B)** Volcano plot illustrating differential gene expression between Scissor+ and Scissor- epithelial cells. Genes with a p-value <0.05 and an average log Fold Change >1 (TATDN1, CAV2, CLDN1, LIPH, MT1E, PSCA, and MMP3) were identified as marker genes for Scissor- epithelial cells. **(C)** Upper: Tuning parameters of OS-related genes were selected to cross-verify the error curve. Lower: The LASSO coefficients for seven genes in the TCGA cohort. Ten-fold cross-validation was applied to calculate the best lambda, which leads to a minimum mean cross-validated error. **(D)** The risk model stratified PAAD patients in the TCGA cohort into high-risk and low-risk groups. **(E)** Kaplan-Meier analysis of OS for PAAD patients in the TCGA cohort, comparing high-risk and low-risk groups. **(F)** The risk model stratified PAAD patients in the ICGC cohort into high-risk and low-risk groups. **(G)** Kaplan-Meier analysis of OS for PAAD patients in the ICGC cohort, comparing high-risk and low-risk groups. **(H)** ROC curve analysis in the TCGA cohort. **(I)** ROC curve analysis in the ICGC cohort. The asterisks represented the statistical P value. *, *P* < 0.05; **, *P* < 0.01; ***, *P <* 0.001; ****, *P* < 0.0001; PAAD, pancreatic adenocarcinoma; OS, overall survival; ROC, receiver operating characteristic; LASSO, least absolute shrinkage and selection operator.

The PAAD patients from the TCGA dataset were categorized into Scissor+ and Scissor- groups based on DEGs. As illustrated in Figure 4A, the Scissor+ group exhibited a poorer prognosis. To further investigate the association between Scissor+ epithelial cells and the prognosis of PAAD patients, LASSO-Cox regression analysis was employed to develop a prognostic risk model, which identified seven genes: TATDN1, CAV2, CLDN1, LIPH, MT1E, PSCA, and MMP3 (Figure 4B). Based on their coefficients, the risk score = expression level of TATDN1 * 0.0383 + expression level of CAV2 * 0.102 + expression level of CLDN1 * 0.0471 + expression level of LIPH * 0.139 + expression level of MT1E * 0.0397 + expression level of PSCA * 0.088 + expression level of MMP3 * 0.055 (Figure 4C). Supplementary Figure S6A showed the correlation analysis between risk model with seven genes. To assess the stability and reliable generalization of the risk model, PAAD patients in the TCGA and ICGC cohorts were separately classified into high-risk and low-risk groups. The high-risk group showed a higher number of deaths (Figures 4D,F). Kaplan-Meier curves indicated that OS, DSS, DFI and PFI were poorer in the high-risk group compared to the low-risk group in the TCGA cohort (Figure 4E; Supplementary Figure S6B,C). Similarly, in the ICGC cohort, PAAD patients in the high-risk group had a worse prognosis than those in the low-risk group (Figure 4G). Additionally, we utilized the ROC curve to evaluate the sensitivity and specificity of this risk model. The results revealed that the AUCs values for 1- and 3-year predictions of OS were 0.74 and 0.81, respectively, in the TCGA cohort (Figure 4H). In the ICGC cohort, the AUCs for 1- and 3-year predictions of OS were 0.56 and 0.64, respectively (Figure 4I). These findings suggest that the risk model has good predictive value in the TCGA cohort.

### 3.5 Nomogram development and validation for PAAD

**FIGURE 5:**
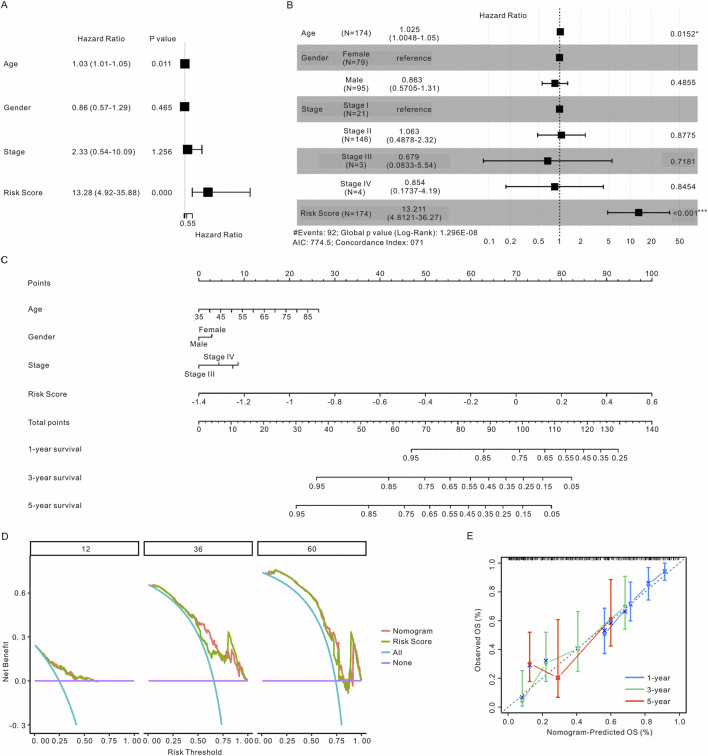
Construction a nomogram model to predict the prognosis of PAAD patients. **(A)** OS related clinical factors identified through univariate Cox regression analysis. **(B)** OS related clinical factors determined by multivariate Cox regression analysis. **(C)** Nomogram integrating clinical factors and risk scores. **(D)** Decision curves comparing the nomogram and risk score alone for predicting 1-, 3- and 5-year outcomes in the PAAD cohort. **(E)** Calibration curve for predicting 1-, 3- and 5-year OS of PAAD patients in the TCGA cohort. PAAD, pancreatic adenocarcinoma; OS, overall survival.

We conducted both univariate and multivariate Cox regression analyses on the TCGA cohort. Our findings indicated that the risk score serves as an independent predictor of OS (Figures 5A,B). Subsequently, we developed a nomogram that incorporates this risk model along with other clinicopathological factors, including age, gender, and tumor stage, to predict the 1-, 3- and 5-year OS in the TCGA cohort (Figure 5C). The predictive performance was evaluated using decision curve analysis and calibration curves. As illustrated in Figures 5D,E, the nomogram demonstrated improved predictive accuracy for the 1-, 3- and 5-year OS of PAAD patients within the TCGA cohort.

### 3.6 Correlation of this risk model with immune microenvironment landscape

**FIGURE 6:**
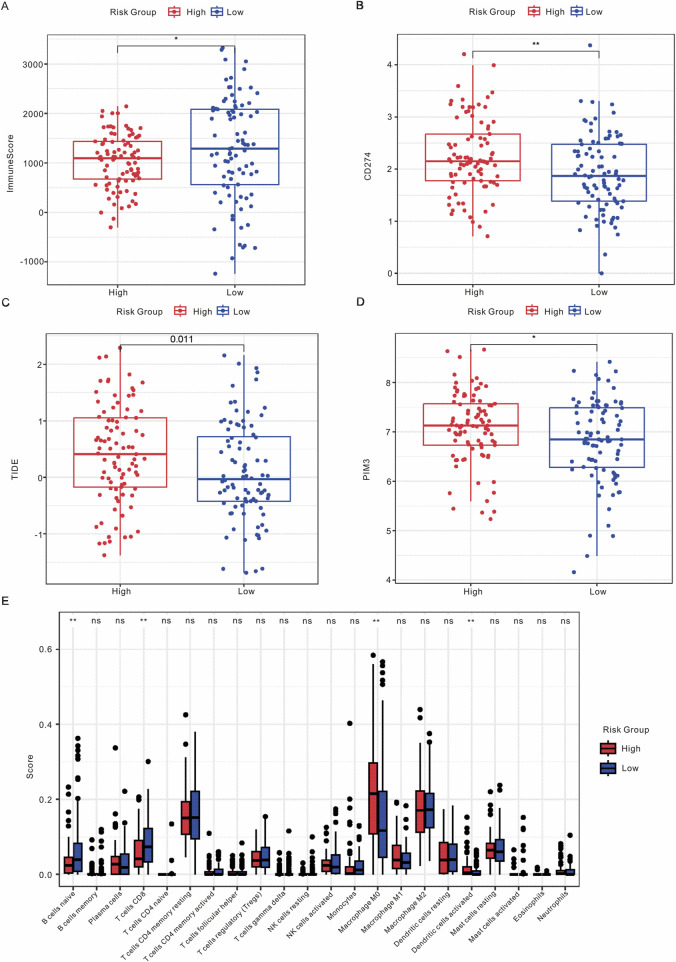
Analysis of the immune microenvironment. **(A)** Comparison of ImmuneScore between high-risk and low-risk groups. **(B)** Expression levels of CD274 in high-risk *versus* low-risk groups. **(C)** Expression levels of TIDE in high-risk *versus* low-risk groups. **(D)** Expression levels of PIM3 in high-risk *versus* low-risk groups. **(E)** Box plot illustrating the distribution of immune infiltrating cells between high-risk and low-risk groups.

To examine the relationship between this risk model and the immune response of tumors, we conducted an analysis investigating the association between the risk model and immune score, stromal score, ESTIMATE score, and tumor purity score. As shown in Figure 6A; Supplementary Figure S7A-C, the low-risk group exhibited a higher immune score, while no significant differences were observed between the risk model and stromal score, ESTIMATE score, or tumor purity score. Additionally, the expression levels of CD274, TIDE, and PIM3 were found to be elevated in the high-risk group (Figures 6B–D). CIBERSORT was employed to analyze the proportions of immune cells, revealing significant differences in naïve B cells, CD8 T cells, M0 macrophages, and activated dendritic cells between the high and low-risk groups (Figure 6E). In addition, we performed the correlation analysis between risk model with immune cells and immune checkpoints. As shown in Supplementary Figure S7D,E, the risk model exhibited a positive association with Macrophage M0, M1, and Dendritic cells, while demonstrating a negative association with CD8^+^ T cells and Mast cells. Furthermore, the risk model was positively correlated with CD74, CD276, CD274, and HAVCR2, and negatively correlated with CTLA4. Overall, these results suggest that Scissor+ epithelial cells may influence metastasis through interactions with the TME.

To evaluate the predictive effect of immunotherapy based on a risk model in tumors, we conducted an analysis of the prognostic outcomes and immunotherapy responses in patients with non-small cell lung cancer (NSCLC) and clear cell renal cell carcinoma (ccRCC) categorized by risk model. The results indicated that NSCLC patients in the high-risk group who received immunotherapy tended to experience disease progression, accompanied by unfavorable progression-free survival (PFS) outcomes (Supplementary Figure S8A-C). Additionally, the rates of progressive disease (PD) and stable disease (SD) were higher in the high-risk group, which was associated with poor OS in ccRCC patients undergoing immunotherapy (Supplementary Figure S8B-D).

Subsequently, gene mutations were analyzed to extract molecular characteristics distinguishing the high-risk and low-risk groups. KRAS, TP53, and CDKN2A exhibited the highest mutation frequencies in the high-risk group, whereas TP53, KRAS, and SMAD4 showed the highest mutation frequencies in the low-risk group (Supplementary Figure S9A,B). Additionally, the TMB was greater in the high-risk group compared to the low-risk group (Supplementary Figure S9C). These findings suggest that the mutation frequencies of the key genes differ between the high-risk and low-risk groups.

### 3.7 The biological effect of LIPH on PAAD cells *in vitro*

**FIGURE 7:**
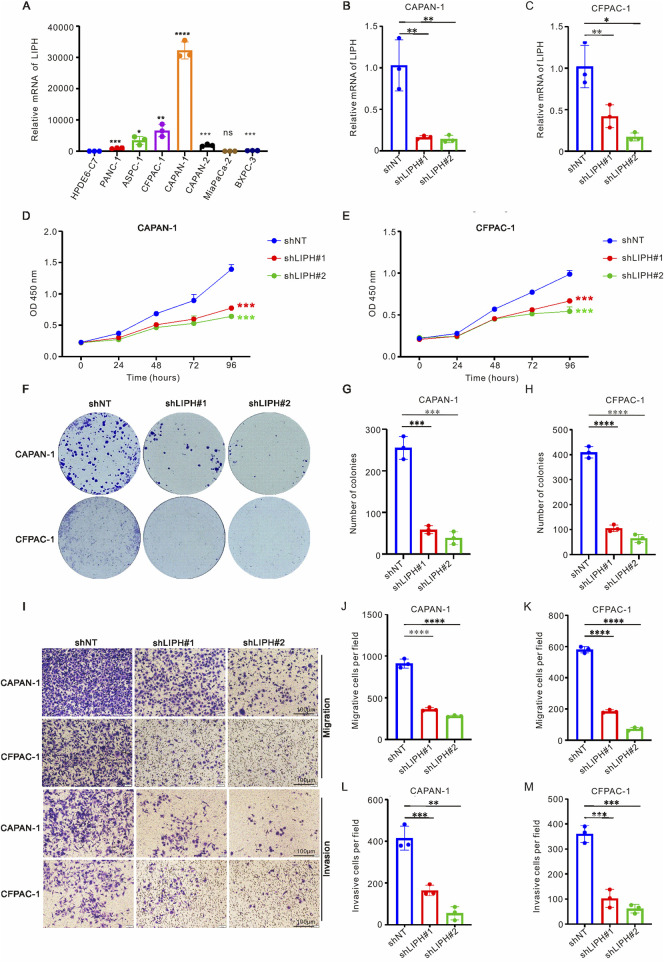
LIPH knockdown inhibited the growth and migration of PAAD cells. **(A)** RT-qPCR was employed to assess the mRNA levels of LIPH in seven PAAD cell lines (PANC-1, ASPC-1, CFPAC-1, CAPAN-1, CAPAN-2, MiaPaCa-2, and BXPC-3) alongside one normal human pancreatic duct epithelial cell line (HPDE6-C7). The relative mRNA levels of LIPH in CAPAN-1 **(B)** and CFPAC-1 **(C)** cells transfected with the shLIPH virus were analyzed. The CCK-8 assay demonstrated that DLAT knockdown significantly inhibited the proliferation of CAPAN-1 **(D)** and CFPAC-1 **(E)** cells. **(F)** Colony formation assays were conducted to evaluate the colony forming ability of LIPH knockdown cells. The number of colonies formed was significantly reduced in CAPAN-1 **(G)** and CFPAC-1 **(H)** cells following LIPH inhibition. **(I)** Transwell assays were performed to assess the migratory and invasive capabilities of LIPH knockdown cells. LIPH knockdown inhibited the migration ability of CAPAN-1 **(J)** and CFPAC-1 **(K)** cells, as well as their invasion ability **(L,M)**. The asterisks represented the statistical P value. *, *P* < 0.05; **, *P* < 0.01; ***, *P* < 0.001; ****, *P* < 0.0001. PAAD, pancreatic adenocarcinoma.

The risk model included seven genes (TATDN1, CAV2, CLDN1, LIPH, MT1E, PSCA, and MMP3), and it remains unclear whether LIPH influences the metastasis of PAAD cells. To investigate the biological effect of LIPH on PAAD cells *in vitro*, we conducted a series of experiments. As demonstrated in Figure 7A, the mRNA levels of LIPH were higher in most PAAD cell lines compared to HPDE6-C7 cells. Consequently, we selected the cell lines CFPAC-1 and CAPAN-1, which exhibited the highest expression levels of LIPH, for further study. We successfully constructed LIPH knockdown cell lines for both CFPAC-1 and CAPAN-1 (Figures 7B,C). CCK-8 assays and colony formation assays revealed that LIPH knockdown significantly inhibited the proliferation and growth of PAAD cells (Figures 7D–H). Additionally, the migration and invasion capabilities were markedly reduced in LIPH knockdown cells (Figures 7I–M). In conclusion, these findings suggest that LIPH promotes the development and invasion of PAAD cells *in vitro*.

## 4 Discussion

Although chemotherapy holds some value for patients with metastatic PAAD, the prognosis remains poor, underscoring the importance of elucidating the underlying molecular mechanisms of pancreatic cancer metastasis. Recent advancements in scRNA-seq have provided unique advantages in exploring tumor and TME heterogeneity, as well as in identifying novel cell subclusters within tumors, which is crucial for the development of clinically relevant therapeutic targets. This study demonstrated the heterogeneity of PAAD and identified eight distinct clusters using published scRNA-seq datasets including epithelial cells, macrophages, T cells, dendritic cells, endothelial cells, fibroblasts, mixed cells, and mast cells. Most of these clusters are involved in regulating tumor metastasis, proliferation, and responses to immunotherapy (Wei et al., 2019; Kalluri, 2016; Verneau et al., 2020). We identified a metastasis-related population of malignant epithelial cells, termed Scissor+ cells, in PAAD by integrating scRNA-seq data with bulk RNA-seq data. We observed two developmental trajectories for malignant epithelial cells transitioning from primary tumors to metastases. Furthermore, we constructed a prognostic risk model for PAAD patients based on Scissor+ epithelial cells, which effectively predicts OS, DSS, DFI and PFI in the TCGA and ICGA cohorts. Additionally, the high-risk group exhibited a significant correlation with elevated expression levels of CD274, TIDE, and PIM3, suggesting that PAAD patients in this group may benefit from immunotherapy. Finally, TATDN1, CAV2, CLDN1, LIPH, MT1E, PSCA, and MMP3 were identified as candidate genes associated with Scissor+ epithelial cells. Our study is the first to reveal that the knockdown of LIPH can inhibit the proliferation and metastasis of PAAD cells *in vitro*. Further investigations are necessary to elucidate the underlying molecular mechanisms.

PAAD predominantly arises from pancreatic ductal epithelial cells, and previous studies have shed light on the heterogeneity of neoplastic epithelial cells during the progression of PAAD (Grant et al., 2016). Kim et al. identified a novel pancreatic cancer cell subcluster, Ep_VGLL1, suggesting that it represents an intermediate subcluster between the basal-like and classical clusters, and confirmed its prognostic value for PAAD patients (Kim et al., 2024). Additionally, Zhang et al. found that CEACAM5+/CEACAM6+ ductal cells are associated with poor prognosis in PDAC patients through scRNA-seq (Zhang S. et al., 2023). Notably, only 22% of basal-like malignant ductal cells influenced the response to chemotherapy and OS of PAAD patients, indicating the heterogeneity present within the intra-tumoral environment and TME (Park et al., 2024). These studies collectively establish that scRNA-seq is a crucial tool for identifying tumor heterogeneity and exploring new subclusters. By analyzing scRNA-seq data, we identified 14 epithelial subclusters and detected malignant epithelial cells in PAAD samples through inferred CNV and aneuploidy analysis. We observed two developmental trajectories for malignant epithelial cells from primary tumors to metastasis. During the metastatic process, the expression of CDH1 decreased while CDH2 increased in malignant tumor epithelial cells, consistent with previous studies that implicate EMT signaling in tumor metastasis (Aiello et al., 2018; Ligorio et al., 2019).

The Scissor algorithm can identify the most phenotype-associated cell subpopulations from single-cell data (Sun et al., 2022; Guccini et al., 2021) and has been applied to lung adenocarcinoma (LUAD) to distinguish cancer cells from normal phenotypes (Lambrechts et al., 2018). Based on the high and low Scissor subtypes of dendritic cells, Cheng et al. constructed a prognostic risk model for esophageal squamous cell carcinoma (Cheng et al., 2024). Additionally, Scissor was utilized to identify epithelial cells associated with the prognostic phenotypes of hepatocellular carcinoma (HCC) patients, lymph node metastasis-related cell subpopulations in LUAD, and endothelial cells linked to the prognostic phenotypes of head and neck squamous cell carcinoma (HNSCC) (Qi and Zhang, 2023; Ji et al., 2023; Yang et al., 2024). In this study, the Scissor algorithm was employed to integrate scRNA-seq with bulk RNA-seq data. The results demonstrated that Scissor+ epithelial cells were identified as malignant epithelial cells and were positively correlated with metastasis in PAAD. A greater number of Scissor+ epithelial cells were observed in primary samples, while more Scissor-epithelial cells were found in metastatic samples. This discrepancy may be attributed to the decrease or evolution of metastasis-associated subsets during the progression of metastasis (Gerstberger et al., 2023). Furthermore, the upregulated genes in Scissor+ epithelial cells were predominantly focused on metastasis-related pathways, such as adhesion, extracellular matrix (ECM) receptors, EMT and TGFβ signaling pathways, which aligns with previous studies (Ho et al., 2020; Wang et al., 2023).

Immunotherapy often uses immune checkpoint inhibitor (ICI) drugs, such as antibodies targeting CTLA-4, PD-1, and PD-L1, which are highly effective. TMB and microsatellite instability (MSI) are indicators that help predict the likelihood of a tumor responding to ICIs. Patients with high TMB or MSI levels often had long-term OS following immunotherapy (Chalmers et al., 2017; Yamamoto et al., 2020). The TME plays a significant role in impacting the effectiveness of ICIs treatments (Samstein et al., 2019). PAAD cells interact with various types of immunosuppressive cells, including tumor-associated macrophages (TAMs), myeloid cells, CAFs and Treg cells, to establish a “cold” TME. This cold environment contributes to resistance against ICIs and results in poor prognosis for PAAD patients (Niu et al., 2024). The strong immunosuppressive microenvironment in PAAD lead to immune evasion and rapid tumor progression (Martinez-Bosch et al., 2018). Different mechanisms were involved in tumor immune evasion. Such as cancer cells decreased immune recognition by downregulating antigen presentation pathways, like the major histocompatibility complex (MHC) I proteins. Reprogramming TAMs in TME to desired phenotypes offers a promising approach for cancer immunotherapy due to its precision and low side effects (Wang et al., 2024). TGFβ signaling in TME suppresses the antitumor functions of various immune cell populations including T cells, resulting immune suppression severely limits the efficacy of ICIs (Derynck et al., 2021). Cytokines are key mediators of cell communication in TME. Some cytokines contribute to host antitumor responses (Propper and Balkwill, 2022). Through the release of cytokines and growth factors, the M2 TAMs in TME establish an immunosuppressive niche in escaping immune detection to inhibit the anti-tumor immunity and drive tumor growth (Mantovani et al., 2017). Previous study examined disulfidptosis-related genes (DRGs) in pan-cancer and indicated the DRGs linked with poor prognosis and predicted responsiveness to immunotherapy (Xu et al., 2025). Yang et al. indicated that PLRN3 mediated M2 macrophage infiltration in various cancers and thus impacted the effectiveness of immunotherapy in LUAD patients (Yang et al., 2025). TMB is a key indicator for predicting immunotherapy response in tumors and individuals with higher TMB levels also have better response rates with immunotherapy (Yu et al., 2021; Li et al., 2020). Xu et al. indicated EPHB2 expression positively correlated with TMB, MSI in pan-cancer and revealed that EPHB2 could improve the predictive effect of immunotherapy responses (Xu et al., 2024). Our analysis also confirmed a positive relationship between the risk model and high TMB, suggesting that high-risk group predict better responses to immunotherapy. In this study, the proportions of naïve B cells, CD8 T cells, M0 macrophages, and activated dendritic cells differed between Scissor+ and Scissor- epithelial cells. Compared to Scissor-epithelial cells, Scissor+ epithelial cells exhibited higher levels of cell-cell communication, particularly with dendritic cells, macrophages, and mast cells, suggesting that Scissor+ epithelial cells may influence the response to immunotherapy. The HLA-LILRB and B2M-LILRB pathways play significant roles in these interactions, consistent with the notion that HLA class I may interact with LILRB1 or LILRB2 to impair the antibody-dependent cellular phago-cytosis function of macrophages (Zeller et al., 2022). LILRB1 and LILRB2 are expressed in various tumor types and are recognized as immunosuppressive markers within the tumor TME (van der Touw et al., 2017). Inhibition of LILRB2 has been shown to enhance T cell activation (Umiker et al., 2023). Furthermore, LILRB2 can interact with HLA-G in the TME, promoting myeloid cell tumor proliferation and increasing tumor immune evasion (Carosella et al., 2021). This study identified the existence of HLA-LILRB pathways between Scissor+ epithelial cells and dendritic cells, macrophages, and mast cells in the TME, mediating the transmission of immune signal inhibition and promoting metastasis. Further investigation into the underlying molecular mechanisms is warranted.

We constructed a prognostic model and identified seven candidate upregulated genes, which included TATDN1, CAV2, CLDN1, LIPH, MT1E, PSCA, and MMP3. TATDN1 is associated with DNA nuclease activity and endodeoxyribonuclease activity; mutations in the TATDN1 gene indicate vascular invasion in HCC patients (Xu et al., 2023). In addition, six copper dependent-related genes including TATDN1were used to perform the prognostic model in PAAD and it indicated that this model can be applied into estimating the prognosis and immunological microenvironment of pancreatic cancer patients (Guan et al., 2022). CAV2 is elevated in various types of tumors and possesses oncogenic properties that promote the progression and metastasis of PAAD cells (Wang et al., 2022). CLDN1, a member of the tight junction protein family, is considered a clinical therapeutic target. It has been reported to enhance CRC stemness and chemoresistance by interacting with EPHA2 (Primeaux et al., 2023). Knockdown of CLDN1 promotes stemness, migration, and invasion of pancreatic cancer cells (Zhu et al., 2024). MT1E has been identified as a tumor suppressor, with its methylation correlating with HCC metastasis (Liu et al., 2020). Additionally, MT1E with other ten genes were used to perform the prognostic model and indicated its prognostic predictive effect for pancreatic cancer patients (Huang et al., 2022). PSCA is upregulated in most cancers, including prostate cancer, bladder cancer, and PAAD. Previous studies have developed human iPSC-derived CAR macrophages and CAR NK cells targeting PSCA, demonstrating strong anti-pancreatic cancer effects in mouse models (Shah et al., 2024). MMP3, a member of the MMP family, has been shown to induce migration of pancreatic cancer cells (Tjomsland et al., 2016). Although Zhuang et al. identified LIPH as a novel unfavorable prognostic biomarker correlated with immunosuppression in pancreatic cancer patients with RNA-seq database, the relationship between LIPH with PAAD metastasis remains unclear (Zhuang et al., 2022). In this study, we first investigated the function of LIPH in PAAD cells. The results confirmed LIPH’s pro-growth and metastatic effects in PAAD cells, thereby laying the foundation for further research into its mechanism of action in the future.

This study has several limitations. Firstly, it relies on previously published scRNA-seq and bulk RNA data, necessitating further validation of the reliability of the prognostic prediction model in future research. The risk model is validated in the TCGA and ICGC cohorts. These two cohorts lack detailed treatment data and have potential biases in survival data. The heterogeneity of PAAD patients should also be considered. Secondly, while we demonstrated that Scissor+ epithelial cells interact with dendritic cells and macrophages through the HLA-LILRB pathways, the specific molecular mechanisms underlying these interactions require further investigation. Additionally, further functional experiments will be necessary to elucidate the biological role of the LIPH gene in PAAD metastasis and to determine whether it can be targeted to enhance the effectiveness of immunotherapies and chemotherapies.

To conclude, the scRNA-seq dataset was utilized to identify the intra-tumor heterogeneity of PAAD, revealing two developmental trajectories for malignant epithelial cells from primary tumors to metastases. We constructed prognostic models based on differentially expressed genes in Scissor+ epithelial cells, demonstrating the significance of this model in predicting the prognosis and immunotherapy response of PAAD patients. Furthermore, the inhibition of LIPH was shown to reduce the metastasis of PAAD cells *in vitro*, suggesting its potential as a marker gene for metastasis-related malignant epithelial cells. This study significantly enhances our understanding of metastasis-specific cell subpopulations and aids in predicting prognosis and immunotherapy outcomes.

## Data Availability

The datasets presented in this study can be found in online repositories. The names of the repository/repositories and accession number(s) can be found in the article/Supplementary Material.
