# Supplementary material for: Constructing Scissor+ risk model to predict prognosis and immunotherapy responses in PAAD by integrating bulk and single-cell RNA sequencing data
Source: Front Pharmacol. 2025 Sep 19;16:1646840. doi: 10.3389/fphar.2025.1646840 (PMC12491314; doi:10.3389/fphar.2025.1646840)
Supplement: Supplementary file 1 [file Supplementaryfile1.docx]

Constructing Scissor+ risk model to predict prognosis and immunotherapy responses in PAAD by integrating bulk and single-cell RNA sequencing data

Gaofei Zhang^1^, Jiao Yu^1^, Fan Zhang^2^, Fang Wang^2^, Congya Zhou^1*^

*** Correspondence:**Congya Zhou
[smant32@163.com](mailto:smant32@163.com)

Supplementary Figures





**Supplementary Figure 1. UMAP and t-SNE visualization of clusters in PAAD patients.** (A) UMAP visualization of clusters from primary and metastatic PAAD patients. (B) t-SNE visualization of clusters from various PAAD patients. (C) t-SNE visualization of clusters from primary and metastatic PAAD patients. (D) t-SNE visualization of PAAD cells differentiated by Seurat cluster coloration. (E) t-SNE visualization of 8 distinct cell types in PAAD patients. (F) t-SNE visualization of marker genes in epithelial cells, immune cells, and matrix cell types. PAAD, Pancreatic adenocarcinoma; UMAP, Unified Flowform Approximation and Projection; t-SNE, t-distributed Stochastic Neighbor Embedding.



 **Supplementary Figure 2. t-SNE visualization of clusters from primary and metastatic PAAD patients.** (A) t-SNE visualization of epithelial cell clusters in PAAD. (B) t-SNE visualization of epithelial cell clusters with aneuploid analysis. (C) t-SNE visualization highlights the epithelial cells in PAAD samples, with Scissor+ epithelial cells indicated in red and Scissor- epithelial cells in blue. PAAD, Pancreatic adenocarcinoma; t-SNE, t-distributed Stochastic Neighbor Embedding.





**Supplementary Figure 3. Related to Figure 2, includes the following components:** (A) The CNV scores across different epithelial cell clusters. (B) The differential trajectories of epithelial cells during the evolution from primary tumors to metastasis, categorized by cellular state. (C) The expression of different genes in epithelial cells derived from primary and metastatic samples. (D) The optimal number of clusters determined by the top 1000 differentially expressed genes in epithelial cells. (E) The results of GO and KEGG analyses for the top 1000 differentially expressed genes in epithelial cells. PAAD, Pancreatic adenocarcinoma; CNV, copy number variant; GO, Gene Ontology; KEGG, Kyoto Encyclopedia of Genes and Genomes.





**Supplementary Figure 4. Related to Figure 3.** (A) The percentage of Scissor+ and Scissor- epithelial cells in primary and metastatic samples. (B and C) Gene sets that are enriched in Scissor+ epithelial cells.





**Supplementary Figure 5. The communications between Scissor+ or Scissor- with other cell types in TME.**



**Supplementary Figure 6. Related to Figure 4.** (A) The correlation analysis between risk scores and seven genes. (B-D) Kaplan-Meier analyses of the DSS, DFI and PFI for PAAD patients categorized into high-risk and low-risk groups within the TCGA cohort. DSS, disease-specific survival; DFI, disease-free interval; PFI, progression-free interval; PAAD, pancreatic adenocarcinoma.





**Supplementary Figure 7. Related to Figure 6.** (A) StromalScore differences between high-risk and low-risk group. (B) PurityScore differences between high-risk and low-risk group. (C) ESTIMATEScore differences between high-risk and low-risk group. (D) The correlation analysis between risk model with immune checkpoint. (E) The correlation analysis between risk model with immune cells.





**Supplementary Figure 8. Prognostic outcomes and immunotherapy responses in NSCLC and ccRCC patients.** (A) Assessment of the immunotherapy response of NSCLC patients related to risk model using GSE135222 dataset. (B) Assessment of the immunotherapy response of ccRCC patients related to risk model using public database (PMID32472114). (C) Assessment of the PFS of NSCLC patients with immunotherapy using GSE135222 dataset. (D) Assessment of the OS of ccRCC patients with immunotherapy using public database (PMID32472114). NSCLC, non-small cell lung cancer; ccRCC, clear cell renal cell carcinoma; PFS, progression-free survival; CR, complete response; PR, partial response; SD, stable disease; PD, progressive disease.





**Supplementary Figure 9. Molecular characteristics of different subgroups.** (A) Top mutated genes in high-risk group. (B) Top mutated genes in low-risk group. (C) The difference of TMB between high-risk and low-risk group. TMB, Tumor mutational burden.
